# Biochemistry and Molecular Biology of Carotenoid Biosynthesis in Chili Peppers (*Capsicum* spp.)

**DOI:** 10.3390/ijms140919025

**Published:** 2013-09-16

**Authors:** María del Rocío Gómez-García, Neftalí Ochoa-Alejo

**Affiliations:** 1 Department of Plant Genetic Engineering, Center for Research and Advanced Studies-Irapuato Unit, National Polytechnic Institute, Km 9.6 libramiento norte carretera Irapuato-León, Irapuato, Guanajuato 36821, México; E-Mail: mrgomez@ira.cinvestav.mx; 2 Department of Biotechnology and Biochemistry, Center for Research and Advanced Studies-Irapuato Unit, National Polytechnic Institute, Km 9.6 libramiento norte carretera Irapuato-León, Irapuato, Guanajuato 36821, México

**Keywords:** *Capsicum*, carotenoids, chili pepper, biosynthesis, regulation

## Abstract

*Capsicum* species produce fruits that synthesize and accumulate carotenoid pigments, which are responsible for the fruits’ yellow, orange and red colors. Chili peppers have been used as an experimental model for studying the biochemical and molecular aspects of carotenoid biosynthesis. Most reports refer to the characterization of carotenoids and content determination in chili pepper fruits from different species, cultivars, varieties or genotypes. The types and levels of carotenoids differ between different chili pepper fruits, and they are also influenced by environmental conditions. Yellow-orange colors of chili pepper fruits are mainly due to the accumulation of α- and β-carotene, zeaxanthin, lutein and β-cryptoxanthin. Carotenoids such as capsanthin, capsorubin and capsanthin-5,6-epoxide confer the red colors. Chromoplasts are the sites of carotenoid pigment synthesis and storage. According to the most accepted theory, the synthesis of carotenoids in chili peppers is controlled by three loci: *c1*, *c2* and *y*. Several enzymes participating in carotenoid biosynthesis in chili pepper fruits have been isolated and characterized, and the corresponding gene sequences have been reported. However, there is currently limited information on the molecular mechanisms that regulate this biosynthetic pathway. Approaches to gain more knowledge of the regulation of carotenoid biosynthesis are discussed.

## 1. Introduction

Carotenoids are widely distributed in nature and are synthesized by all photosynthetic organisms (cyanobacteria, algae and plants) as well as non-photosynthetic microorganisms, such as fungi and some bacteria [1]. Since the first independent reports elucidating the structure of β-carotene by Richard Kuhn and Paul Karrer in 1928–1930, more than 700 natural carotenoids had been described prior to 2004 [2]. It is estimated that the annual worldwide carotenoid production by nature is equivalent to approximately 100 million tons [3], and more than 20 new structures are reported each year [2].

Carotenoids are isoprenoid compounds that consist of eight isoprene (ip) units attached in a head-tail pattern where the double bond order is inverted at the center of the molecule [4]. Carotenoids comprise a wide group of lipophilic pigments that provide a series of colors, including yellow, orange and red. These pigments are responsible for the coloring of many flowers such as marigolds [5], daffodils, *Gentiana lutea* and *Sandersonia aurantiaca* [6]; fruits such as tomatoes [7], papayas [8], mandarins and oranges [9]; and roots (carrots) [10]. Carotenoids are mostly found intracellularly at the chloroplast and chromoplast membranes in plants. Traditionally, they have been structurally classified as carotenoids, including α-carotene, β-carotene and xanthophylls such as β-cryptoxanthin, lutein, zeaxanthin, violaxanthin, neoxanthin and fucoxanthin [4]. Chromophore length determines the absorption spectrum of a carotenoid molecule and thus its color to the eye [11].

The main function of carotenoids is the protection of cells and organelles against oxidative damage, which they accomplish by interacting with singlet oxygen molecules and scavenging peroxy radicals, thus preventing the accumulation of harmful oxygen species [12]. They are also involved in photosynthesis (participating in the light-harvesting process and as photoprotectors of the photosynthetic apparatus), within the xanthophyll cycle (protecting against light damage) [4], and as precursors of abscisic acid. Additionally, carotenoids have a paramount ecological function because they act as attractants for pollinators and seed dispersal agents [13]. Furthermore, oxidative cleavage of carotenoids by a family of carotenoid cleavage dioxygenases (CCDs; enzymes that cleave double bonds) leads to the production of apocarotenoids, compounds with a variety of biological important activities such as phytohormones (ABA, and strigolactones, a group of terpene lactones with hormone activity that promote germination of root parasitic plants, stimulate symbiotic interactions between plants and arbuscular mycorrhizal fungi, and inhibit shoot axillary branching), the visual and signaling molecules retinal (chromophore of various visual pigments in animals) and retinoic acid (nuclear receptor ligand that is a major signal controlling a wide range of transcriptional processes), and the aromatic volatiles β-ionone (pollinator attractant and fruit or vegetable flavor), β-cyclocitral, geranial, geranyl acetone, theaspirone, α-damascenone and β-damascenone responsible for the flavor and aroma/scent of a number of flowers and a diversity of foods [14–16].

Carotenoids have an essential function in human nutrition and health; humans are unable to *de novo* synthesize vitamin A from endogenous isoprenoid precursors, but plant carotenoids (β-carotene, α-carotene, γ-carotene and β-cryptoxanthin; provide the primary dietary source of provitamin A (meaning they can be converted into retinol) [17]. In addition to their nutritional value, carotenoids, acting as antioxidants, have been implicated in reducing the risk of cancer and cardiovascular diseases [18]. α- and β-carotene suppress tumorigenesis in the skin, lung, liver and colon [19]. Lycopene prevents cardiovascular diseases and possibly prostate cancer [20]. Likewise, it was reported that a diet rich in carotenoids is directly connected to a reduced risk of age-related macular degeneration [21]. Similarly, zeaxanthin and lutein (essential components of the macular pigment in the eye) showed the strongest association between dietary intake and reduced risk of macular degeneration [1,22]. Apocarotenoids have also showed interesting multifunctional activities, and can be useful in the prevention of cancer and other degenerative diseases [17]. Chili pepper fruits produce and accumulate apocarotenoids such as apo-14′-zeaxanthinal, apo-13-zeaxanthinone, apo-12′-capsorubinal, apo-8′-capsorubinal, 9,9′-diapo-10,9′-*retro*-carotene-9,9′-dione, apo-8′-zeaxanthinal, apo-10′-zeaxanthinal, apo-12′-zeaxanthinal, apo-15-zeaxanthinal, apo-11-zeaxanthinal and apo-9-zeaxanthinone [23].

At the commercial level, carotenoids have a broad variety of applications, such as nutrient supplements, for pharmaceutical purposes, in animal feeds [12], coloring foods, cosmetics and nutraceutical agents as well [4].

## 2. *Capsicum* Fruit Carotenoids

**Figure 1:**
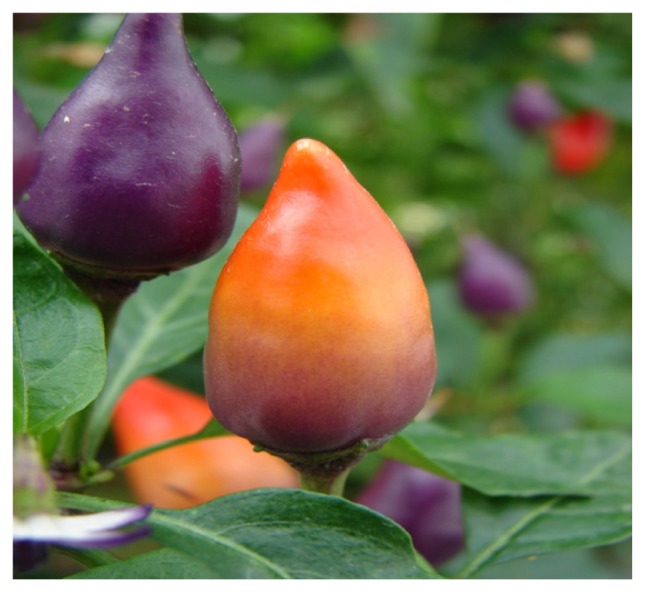
Chili pepper fruits (*Capsicum annuum*; “Bolivian”) showing carotenoids and anthocyanins in the pericarp tissue.

Chili pepper fruits synthesize and accumulate a variety of compounds, such as the characteristic capsaicinoids (hot compounds), vitamins (Vitamins A, C and B), and pigments (anthocyanins and carotenoids) (Figure 1). Studies of *Capsicum* fruit carotenoid pigments began in the nineteen-century. Henri Braconnot reported the first investigations of *Capsicum annuum* (paprika) pigments in 1817 [24]. One pigment from *Capsicum annuum* (paprika) was obtained in a crystalline form in 1927, and the name capsanthin was proposed [25]. Similarly, von Zechmeister and von Cholnoky [26] published a series of articles on the carotenoid content of paprika and found carotenoids such as β-carotene, cryptoxanthin and zeaxanthin that had not been previously described, in addition to capsanthin and capsorubin [27]. Other new carotenoids from paprika, such as lutein epoxide, antheraxanthin, violaxanthin, cryptocapsin, and mutatoxanthin, were also reported [28]. Later, it was found that capsanthin and capsorubin contained one and two cyclopentane rings, respectively, adjacent to their keto groups, which were part of the conjugated double bond system [29,30].

## 3. Carotenoid Content and Composition during Chili Pepper Fruit Ripening

Chili pepper fruits undergo profound morphological, physiological and metabolic transformations in terms of pigment composition and content during ripening. These changes in fruit composition are affected by the genotype, maturity and growth conditions [31,32]. Chlorophyll is responsible for the green color of chili pepper fruits, anthocyanins are violet/purple pigments, and the yellow-orange colors are provided by α- and β-carotene, zeaxanthin, lutein and β-cryptoxanthin. Carotenoids with oxygenated forms such as those bearing acylcyclopentanol end groups control the red color in chili pepper fruits. The most characteristic examples are capsanthin, capsorubin and capsanthin-5,6-epoxide [33].

Capsanthin is a red color pigment that is lipophilic and is mostly synthesized and accumulated in red *Capsicum* spp. fruits but also in *Lilium*, *Aesculus* and *Berberis* species and in fruits from *Asparagus officinalis* [34–42]. Capsanthin levels may represent up to 50% of total carotenoid fruit content during the ripening stage. The capsanthin structure contains 11 conjugated double bonds, a conjugated keto group and a cyclopentane ring. Due to these characteristics, capsanthin is a powerful antioxidant (good radical scavenger) [43]. Though β-carotene and β-cryptoxanthin possess the same number of double bonds as capsanthin, their antioxidant abilities are lower than that of capsanthin. Although capsanthin does not have provitamin A properties, it is considered a functional compound due to its antioxidant and anti-tumor (colon cancer) activities [44].

Carotenoids are not randomly dispersed or freely accessible in chromoplasts, but rather 95% of these pigments accumulate in specific substructures: the fibrils [45]. These fibrils are aligned along the longitudinal axes of chromoplasts, which explains their birefringence. Purified fibrils contain galactolipids, phospholipids and a single 32 kDa protein named fibrillin. A model for the chromoplast fibril architecture proposes that carotenoids accumulate in the center of the fibrils and are surrounded by a layer of polar lipids (galactolipids and phospholipids), which in turn are surrounded by an outer layer of fibrillin that is directly attached to the plastid stroma [45].

The content and composition of the carotenoid profiles in chili pepper fruits during ripening is determined by two metabolic processes: (1) transformation of existing photosynthetic pigments; and (2) *de novo* carotenoid biosynthesis. For example, studies of the pigment content during several ripening stages (green, color break I, color break II, red I, red II), in the chili pepper cultivars Bola and Agridulce revealed that the chlorophyllic pigments (lutein and neoxanthin) disappeared in the red I and II stages (mature fruit), whereas the concentrations of intermediates in the synthesis of red pigments (β-carotene and violaxanthin) increased, and zeaxanthin, capsanthin, capsorubin, β-cryptoxanthin and capsolutein were synthesized *de novo* [46]. However, in yellow-orange chili pepper fruits from *C. baccatum*, *C. pubescens* and *C. annuum*, violaxanthin was the major carotenoid (37% to 68% of total carotenoids), followed by *cis*-violaxanthin, antheraxanthin and lutein (5% to 14%) [47].

Rates of carotenoid accumulation in mature red chili pepper fruits (*C. annuum*) have often been found to be as follows, in decreasing order: ketoxanthophylls (capsanthin, capsorubin and capsolutein), xanthophylls (zeaxanthin, neoxanthin and violaxanthin), epoxyxanthophylls (capsanthin-5,6-epoxide and capsanthin-3,6 epoxide) and hydrocarbons (lycopene) [48].

In ripe chili pepper fruits, most carotenoids are esterified with fatty acids, making them liposoluble [49] and facilitating their accumulation in the lipophilic globule at the chromoplasts [44]. The esterification process takes place mostly in *de novo* biosynthesized pigments such as capsanthin, capsorubin, zeaxanthin and β-cryptoxanthin, although it also occurs in previously synthesized pigments such as violaxanthin [50]. *De novo* biosynthesis and esterification occur simultaneously during chili pepper fruit ripening. Red xanthophylls, such as capsanthin, are esterified by short chains of saturated fatty acids such as lauric (12:0), myristic (14:0) and palmitic acid (16:0), while yellow xanthophylls are mainly esterified by myristic (14:0), palmitic (16:0) and an unsaturated linoleic acid form (18:2^Δ9,12^). For this reason, the higher numbers of double bonds in the fatty acid chains in yellow xanthophylls make them less stable compared to their red counterparts [51]. Similarly, in full ripe chili pepper fruits (*Capsicum annuum* cv. Bola), the free carotenoids and partially and totally esterified forms of these pigments account for 21.3%, 35.6% and 43.1% of the total, respectively [51]. Likewise, the balance among these three esterified fractions in fully ripe chili pepper fruits (*C. annuum*) appears to be well conserved in Numex, Mana, Belrubi, Negral and Delfin cultivars, and these might be used as a “ripeness index”. Interestingly, the “Negral” chili pepper, which retains chlorophyll at the fully ripe stage, showed the same degree of esterification as other red chili pepper fruit cultivars, supporting the idea that the carotenogenesis process is independent from the chlorophyll catabolism [50].

Total carotenoid content in mature red chili pepper fruits can increase from two up to 60 times compared to the immature fruits. For instance, in black paprika fruits (*C. annuum*), the total carotenoid content during ripening ranges from 48.5 mg/100 g (d.wt.) in immature green fruits up to 3211 mg/100 g in mature red fruits, which represents a 66-fold increase (Table 1) [54]. Carotenoid composition also undergoes drastic changes during ripening. In general, the most abundant carotenoids in immature green chili pepper fruits are lutein, β-carotene and neoxanthin; their percentages in green bell peppers are: lutein (40.8%), neoxanthin (15.1%) and β-carotene (13.4%) (Table 1) [57]. However, carotenoid composition changes as fruit ripens; for example, in red bell peppers, the main carotenoids are capsanthin (35%), β-carotene (11.6%) and violaxanthin (10%) (Table 1) [27]. These modifications to carotenoid composition have been observed in other chili pepper varieties as well (Table 1). Of the total carotenoid content (9.15 mg/100 g f.wt.) in the Signal Red variety (*C. annuum*), capsanthin (predominant pigment) represented 46% [57]. Likewise, the total carotenoid content is also dependent on variety. Generally, “Signal Red” > “Signal Orange” ≈ “Signal Yellow” ≈ “Signal Green”, and red pepper fruits have the highest antioxidant activity.

A detailed study of the relative carotenoid content in Red paprika (*Capsicum annuum* var. *lycopersiciforme rubrum*) at six ripening stages was described [55]. Thirty-five different known carotenoids varied with the developmental stage. Lutein was the most abundant in the green stage, then decreased with further ripening stages and was absent in the red or deep red stages. Capsanthin levels were low in the early stages and increased gradually until the red stage, at which the highest content was recorded. β-carotene was almost constant throughout the growth and ripening of fruits.

In mature yellow pepper fruits, the carotenoid content and composition is quite different as compared to the red fruits. Violaxanthin (34%), antheraxanthin (10.5%), lutein (9.2) and zeaxanthin (8.5) were the most abundant carotenoids in immature (green) yellow pepper fruits. These fruits were devoid of red carotenoids (capsanthin, capsorubin and cryptocapsin) [54].

Chili pepper fruits from some cultivars are capable of retaining chlorophyll (delayed chlorophyll senescence) at the fully ripening stage, producing a dark-brown color due to the combination of chlorophyll and red carotenoids [58]. For example, “Negral” retained chlorophyll at the fully ripe stage (almost 14% of chlorophylls at the green stage), whereas “Bola Roja” fruits reached the fully ripe stage without any chlorophyll [59]. These differences between chili pepper varieties or cultivars are attributed to differences in the plastid ultrastructure. The persistence of a long peripheral thylakoids in the chromoplasts of “Negral” chili peppers was most likely associated with the presence of chlorophyll in fully ripe fruits of this cultivar. This study concluded that “Negral” chili peppers have undergone a minor plastid evolution compared to their “Bola Roja” counterparts [59].

Analyzing the changes in the accumulation of individual carotenoids in red fruit chili peppers in different cultivars (*Capsicum annuum* L. “Mana”, “Numex”, “Belrubi”, “Delfin” and “Negral” (a chlorophyll-retaining mutant at the ripe stage)) during fruit ripening has revealed an inverse relationship between total carotenoid content and the red to yellow isochromic pigment fraction ratio (R/Y) as well as the capsanthin-to-zeaxanthin ratio (Caps/Zeax) [50]. This relationship appears to be related to the carotenogenic capacity of the cultivar, and thus selection and breeding should not only seek higher total carotenoid content but also attempt to increase these ratios. For example, the Mana cultivar had the highest total carotenoid content (13,208 mg/kg d.wt.) but the lowest R/Y (1.25) and Caps/Zeax (3.38) ratios, and therefore these are the parameters to improve. Cultivar Negral had a high carotenoid content (8797 mg/kg d.wt.) and high R/Y and Caps/Zeax ratios as well, and thus it could be used to transfer these characteristics into Mana cultivar by direct crosses. Interestingly, chili pepper fruits undergo substantial structural changes during the late ripening stages [60]. In general, chloroplasts developed into chromoplasts, which are rich in carotenoids. The typical chloroplast thylakoid structure disintegrates and is replaced by non-chlorophyllous single thylakoids, which are derived in part from the inner envelope membrane. These structural changes correlate with an enhanced accumulation of keto-carotenoids (capsanthin, capsorubin and cryptocapsin). Lipid metabolism is affected as well, as galactolipid levels are diminished while phospholipids accumulate.

Carotenoid composition analysis of red paprika fruits (*C. annuum*, var. *lycopersiciforme rubrum*) during ripening showed that the major component was capsanthin (37%), but numerous minor carotenoids were also present. These included violaxanthin, antheraxanthin and capsanthin-5,6-epoxide (5,6-epoxy-end group); capsanthin-3,6-epoxide and cucurbitaxanthin B (3,6-epoxy end group); 5,6-diepikarpoxanthin (3,5,6-trihydroxy end group); capsanthone (3-oxo-κ end group); nigroxanthin (γ end group); and capsorubin, cryptocapsin and several furanoid oxides and *cis* isomers [55]. A possible biosynthetic pathway for the formation of minor carotenoids containing 3,5,6-trihydroxy-β, 3,6-epoxy-β, and 6-hydroxy-γ end groups was proposed.

Recently, a study of red, orange and yellow chili pepper fruits from 28 cultivars (*C. annuum* (24), *C. frutescens* (1) and *C. chinense* (3)) was conducted to examine the relationship between fruit color and β-carotene content, to characterize the content of six carotenoids in orange-fruited varieties and to analyze the DNA sequences of four structural biosynthetic genes to identify metabolic and genetic differences between them [61]. The most abundant carotenoids in pericarp tissues were β-carotene, capsanthin and capsorubin. A large degree of variability in terms of β-carotene, capsanthin and total carotenoids was observed in all cultivars. Orange color was mainly determined by the presence of β-carotene but in two cases was due to the accumulation of red and yellow carotenoids. Examination of phytoene synthase (*PSY*), lycopene β-cyclase (*LCY-B*), a β-carotene hydroxylase (*BCH; CRTZ-2*) and capsanthin-capsorubin synthase (*CCS*) gene sequences indicated that different alleles for specific carotenoid biosynthetic enzymes were associated with the accumulation of specific carotenoids in orange fruits that could be used as molecular markers for high β-carotene (high provitamin A) fruit content selection.

The antioxidant activities of chili pepper fruits are also affected by ripening. Fully ripe chili pepper fruits exhibit higher antioxidant activity than green fruits. This difference is easily explained by the increased amounts of carotenoids, phenolics, flavonoids and ascorbic acid that are present in fully ripe chili pepper fruits [33]. The levels of these antioxidants in chili pepper fruits may be affected by genotype, plant growth conditions and post-harvest management [62].

Finally, in non-photosynthetic organs such as seeds, flowers or red fruits, carotenoids have complex structural diversity, as these compounds undergo a series of secondary metabolic reactions such as oxidation, cleavage of polyene chains, epoxidation, and (*Z*/*E*) (*cis*-*trans*) isomerization, among others. Therefore, novel carotenoid structures are detected in these plant organs [44]. The improvement of analytical instruments such as NMR, MS and HPLC has made it possible to determine the structures of very minor carotenoids found in nature. For instance, the new carotenoid compounds 3′-deoxy-capsanthin and 3,4-dehydroxy-3′-deoxycapsanthin, which are minor components of mature paprika fruits (*C. annuum*), were isolated and characterized [63]. Their chemical structures were determined to be (3*R*, 5′*R*)-3-hydroxy-β,κ-caroten-6′-one and (5′*R*)-3,4-didehydro-β,κ-caroten-6′-one, respectively, using UV-vis, NMR, CD (Circular Dichroism), HRFABMS (High Resolution Fast Atom Bombardment Mass Spectrometry) and FABMS (Fast Atom Bombardment Mass Spectrometry)/MS spectra. These compounds are the first examples of carotenoids that possess a 6-oxo-κ end group.

## 4. Carotenoid Biosynthesis Pathway in Plants

The carotenoid biosynthesis pathway in plants is tightly linked to other biosynthetic routes such as those for gibberellins, tocopherols, chlorophylls and phylloquinones via the five-carbon (C5) compound isopentenyl pyrophosphate (IPP). Since the discovery of the mevalonate (MVA) pathway in the 1950s, it was assumed that IPP was synthesized from acetyl-CoA via mevalonate [3]. Although the MVA pathway may contribute to carotenoid biosynthesis in some cases, such as in etiolated seedlings [22], plant carotenoids are mainly produced through the methylerythritol phosphate (MEP)-derived pathway in light-grown plants [64–66].

**Figure 2:**
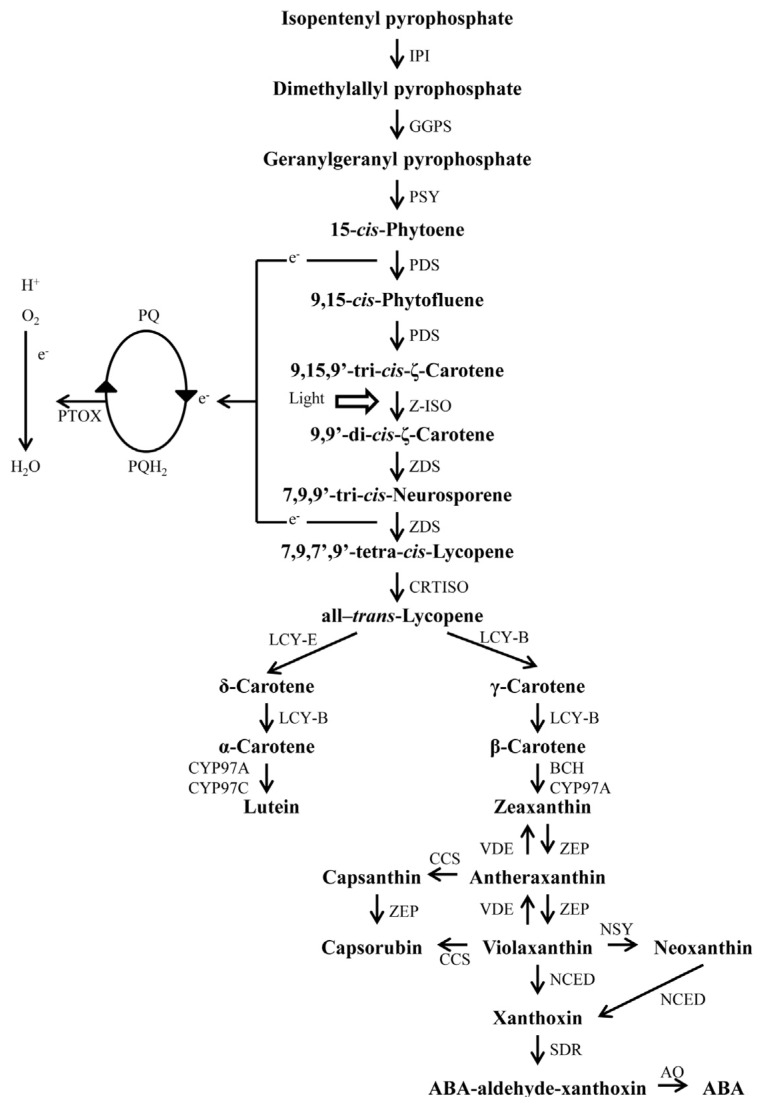
Carotenoid biosynthesis pathway in plants. IPI, isopentenyl pyrophosphate isomerase; GGPS, geranylgeranyl pyrophosphate synthase; PSY, phytoene synthase; PDS, phytoene desaturase; Z-ISO, ζ-carotene isomerase; ZDS, ζ-carotene desaturase; CRTISO, carotene or carotenoid isomerase; LCY-B, lycopene-β-cyclase; LCY-E, lycopene-ɛ-cyclase; BCH, β-carotene hydroxylase or carotene β-hydroxylase (non-heme di-iron type); CYP97A, β-carotene hydroxylase (cytochrome 450 type); CYP97C, ɛ-carotene hydroxylase (cytochrome 450 type); ZEP, zeaxanthin epoxidase; CCS, capsanthin-capsorubin synthase; VDE, violaxanthin de-epoxidase; NSY, neoxanthin synthase; NCED, 9-*cis*-epoxycarotenoid dioxygenase (a carotenoid cleavage dioxygenase; CCD); SDR, short-chain dehydrogenase/reductase; AO, aldehyde oxidase; ABA, abscisic acid; PTOX, plastid terminal oxidase; PQ, oxidized plastoquinone; PQH_2_, reduced plastoquinone. Adapted from [69–77].

All plant carotenoids are synthesized from units of five carbon compounds, IPP and its isomer dimethylallyl pyrophosphate (DMAPP) (Figure 2). The addition of three IPP molecules to a DMAPP molecule generates geranylgeranyl pyrophosphate (GGPP), the immediate precursor of carotenoids. This reaction is catalyzed by GGPP synthase (GGPS) [1]. This enzyme was isolated as a homodimer (74 kDa) from chili pepper chromoplasts [67], and its corresponding cDNA was identified and sequenced [68]. GGPP is the common precursor for various plastid isoprenoids, including gibberellins, chlorophylls, tocopherols, phylloquinones, plastoquinones and carotenoids [1].

The first step in plant carotenoid biosynthesis pathway is the formation of phytoene (Figure 2); this is a two-step condensation reaction with two GGPP molecules catalyzed by PSY [1]. Phytoene synthesis in the stroma of chloroplasts, etioplasts and amyloplasts from tissues of different plant species (*Capsicum annuum*, *Pisum sativum*, *Spinacia oleracea*, *Hordeum vulgare*, *Triticum aestivum* and *Zea mays*) was first reported by Dogbo *et al.* [78]. PSY biochemical characterization was carried out using chili pepper tissues [79]. The phytoene molecule is made of a 40-carbon isoprenoid polyene chain with conjugated double bonds that form the backbone of plant carotenoids and determine their physical and biological properties [1]. The second step is the formation of lycopene, which occurs after four phytoene desaturation and isomerization reactions leading from 15-*cis*-phytoene to all-*trans*-lycopene (Figure 2). These desaturations and isomerization reactions sequentially produce 9,15-*cis*-phytofluene, 9,15,9′-tri-*cis*-ζ-carotene, 9,9′-di-*cis*-ζ-carotene, 7,9,9′-tri-*cis*-neurosporene and finally all-*trans*-lycopene (Figure 2) [77] and increase the conjugated double bond series, which constitutes the chromophore, transforming non-colored phytoene into red lycopene [80]. In plants, the four desaturation reactions are catalyzed in two steps by two phylogenetically related enzymes: phytoene desaturase (PDS) and ζ-carotene desaturase (ZDS). The enzyme responsible for the desaturation of phytoene to ζ-carotene is PDS. Phytoene desaturase genes were identified and isolated from chili peppers [81]. Phytoene (15-*cis*-phytoene) suffers two sequential desaturations by PDS to produce 9,15-*cis*-phytofluene and 9,15,9′-*cis*-ζ-carotene, which can be isomerized into ζ-carotene by light (Figure 2). However, carotenogenesis in organs under dark conditions, such as roots and etiolated leaves, suggests the participation of a ζ-carotene isomerase (Z-ISO) that transforms 9,15,9′-tri-*cis*-ζ-carotene into 9,9′-di-*cis*-ζ-carotene (Figure 2) [76,77,82]. Recently, a *Z-ISO* gene from maize and *Arabidopsis* was isolated and characterized and was found to be important for both light-exposed and tissues under dark conditions [83]. ZDS catalyzes two desaturation steps of 9,9′-di-*cis*-ζ-carotene to produce 7,9,9′-tri-*cis*-neurosporene, and 7,9,7′9′-tetra-*cis*-lycopene; and finally, a carotenoid isomerase (CRTISO) converts this later compound into all-*trans*-lycopene (Figure 2) [76,77]. A cDNA encoding the gene for ZDS was cloned from chili peppers and expressed in *E. coli* [84]. The deduced amino acid sequence of this cDNA encoded a protein with a mature size of approximately 59 kDa derived from a precursor polypeptide with a NH_2_-terminal extension resembling transit peptides for plastid targeting.

In *Arabidopsis* etioplasts and tomato chromoplasts, carotenoid isomerase (CRTISO) activity is necessary to transform pro-lycopene (product of PDS and ZDS activities) into lycopene [85,86]. However, it appears that photo-isomerization can substitute for CRTISO in chloroplasts. Thus, it is possible that CRTISO in plants functions in carotenoid biosynthesis in non-photosynthetic tissues [1]. More recently, two CRTISO (*Zmcrtiso1* and *Zmcrtiso2*) cDNAs were identified from corn (*Zea mays*) mapping to different chromosomes [76]. ZmCRTISO1 exhibited activity on tetra-*cis*-prolycopene and produced all-*trans*-lycopene, but was not capable of acting on the 15-*cis* double bond of 9,15,9′-tri-*cis*-ζ-carotene; ZmCRTISO2 was inactivated by a premature termination codon in the inbred line B73, but the mutation was absent in other corn cultivars and the activity was the same as ZmCRTISO1.

It has been proposed that phytoene desaturation involving PDS and ZDS requires the transfer of two electrons to oxidized plastoquinones, which in turn are re-oxidized by a plastid terminal oxidase (PTOX) using O_2_ as a terminal acceptor (Figure 2) [70]. Mutant plants affected in *PTOX* gene (*immutants* and *ghost*) are defective in carotenoid production and accumulate phytoene; as a result, these mutants exhibit photobleaching in high light conditions because of a lack of photoprotective carotenoids [69,70,87–90]. PTOX is a plastid-located plastoquinol:oxygen oxidoreductase linked to carotenoid biosynthesis and chlororespiration to prevent photooxidative damage [91]. PTOX is synthesized as a precursor polypeptide, which is imported into chloroplasts and inserted into the thylakoid membrane [69]. In *Arabidopsis* a *PTOX* cDNA showing a 350-amino acid ORF encoding a putative 40.5 kD protein was isolated [69]. The genomic sequence of PTOX revealed a structure composed of nine exons and 8 introns [69].

The third step in carotenoid biosynthesis is the cyclization of lycopene (Figure 2). Carotenoid cyclization is limited to the formation of a six-membered ring at one or both ends of the acyclic precursor. These end groups merely differ in the position of a double bond in the cyclohexane ring. The type of end group depends on the nature of the cyclase enzyme [3]. The mechanism of cyclization involves a proton attack at C2 and C2′ of lycopene. The resulting carbonium ion intermediate is stabilized by loss of a proton from either C1 or C4 to yield a β- or є-ring, respectively [55]. The formation of β-carotene is catalyzed by a LCY-B, which adds a β ring to each final end of lycopene to produce β-carotene. In the case of α-carotene, the lycopene є-cyclase (LCY-E) attaches a ɛ ring to lycopene and generates δ-carotene; then, LCY-B joints a β ring to the final end of δ-carotene to yield α-carotene (Figure 2) [1]. LCY-B has been isolated from a variety of red paprikas [92,93].

The fourth step in the carotenoid biosynthesis pathway is a hydroxylation step (Figure 2). Xanthophylls are oxidation products derived from α and β-carotene. Zeaxanthin and lutein are formed by hydroxylation at the 3 and 3′ carbon atoms of β,β-carotene or β,є-carotene, respectively, by separate hydroxylases, each one specific for β or є rings [55]. β,β and β,ɛ xanthophylls are produced by two different classes of carotene hydroxylases: heme-(cytochrome P450) (CYP97) and non-heme (di-iron) (BCH; also abbreviated β-CH, CHY, CHYB, CRTR-B, HYD or HYDB by different authors) (Figure 2) [94]. Hydroxylation of the two β-ionone rings in β-carotene generates zeaxanthin, whereas hydroxylation of the one β-ring and one ɛ-ring in α-carotene leads to lutein formation. Hydroxylation of β-rings in the carotenes is potentially mediated by either the P450-type CYP97A or di-iron β-CH enzymes, while hydroxylation of the ɛ-ring of α-carotene is carried out by CYP97C, another P450 enzyme. Zeaxanthin is easily converted into violaxanthin, via antheraxanthin, introducing the 5,6-epoxy group to the 3-hydroxy β-rings. This reaction is catalyzed by a zeaxanthin epoxidase (ZEP) (Figure 2). Conversely, in leaves growing under high light intensity, violaxanthin de-epoxidase (VDE) catalyzes the de-epoxidation reaction in two steps, allowing the transformation of violaxanthin into zeaxanthin (this reverse reaction is much more efficient at dissipating the excess excitation energy) (Figure 2). However, when light conditions return to normal values, zeaxanthin is transformed into violaxanthin. This reversible inter-conversion is known as the xanthophyll cycle and is of key importance for NPQ (non-photochemical quenching) and for plant adaptation to changes in environmental conditions [95].

The last step in carotenoid biosynthesis is the conversion of violaxanthin into neoxanthin by the action of a neoxanthin synthase (NSY; also abbreviated NXS by some authors) (Figure 2) [1,71]. Violaxanthin can also be converted into xanthoxin and subsequently into abscisic acid through the action of a 9-*cis*-epoxycarotenoid dioxygenase (NCED) and an aldehyde oxidase (AO) (Figure 2). It has been proposed that neoxanthin can be also converted into xanthoxin by the NCED (Figure 2) [71,72].

## 5. Carotenoid Biosynthesis in Chili Pepper Fruits

*Capsicum* has been one of the most important models to study the chemistry and biosynthesis of carotenoids in plants. Chili pepper fruits accumulate yellow, orange or red carotenoids during the ripening process. Chili pepper fruits exhibit green color due to the presence of chloroplasts during the early stages, but these organelles suffer profound modifications during the late fruit ripening stages to become chromoplasts, which are carotenoid-accumulating plastids [60].

Labeling experiments demonstrated that *Capsicum* chromoplasts incubated with labeled isopentenyl pyrophosphate synthesized several labeled carotenes, among them phytoene, *cis*-phytofluene and *trans*-phytofluene, β-carotene and ζ-carotene [96]. However, α-carotene was poorly labeled. These results demonstrated that chromoplasts are the site of carotenoid biosynthesis and that those carotenoids derived from β-carotene are predominantly synthesized during fruit ripening. Moreover, phytoene is biosynthesized in the stroma, and the membrane fraction (chromoplast envelope plus achlorophyll lamellae derived in part from the inner envelope membrane) is the site where desaturation and cyclization reactions occur during the biogenesis of colored carotenoids. A protocol for the isolation and purification of *Capsicum annuum* chromoplasts was reported [97]. In summary, the lipid composition, carotenoid content and the incorporation of [1-^14^C]isopentenyl pyrophosphate into carotenoids in purified chromoplasts has been described.

Xanthophylls are oxidation-derived products of carotenoids. In *Capsicum*, xanthophylls are responsible for the yellow, orange and red colors in fruits [57]. The keto-xanthophylls (capsanthin, capsorubin and cryptocapsin) are found in red pepper fruits synthesized in the same way as β-carotene via a rearrangement of the epoxy-cyclohexenyl groups of β,β-xanthophylls (antheraxanthin and violaxanthin) (Figure 2) [98,99]. The suggested mechanism involves a pinacolic rearrangement. Conversion of epoxy-xanthophylls (antheraxanthin and violaxanthin) into keto-xanthophylls (capsanthin and capsorubin) is carried out at the *Capsicum* chromoplast membranes (Figure 2) [100].

A metabolic correlation analysis for a number of carotenoids including β-carotene, β-cryptoxanthin, cryptocapsin, capsanthin, capsorubin, neoxanthin, mutatoxanthin and luteoxanthin was carried out by means of a Pearson matrix calculated from the observed pigment concentrations in two *Capsicum* cultivars (Bola Roja and Negral) during fruit maturation as an approach to investigate the participation of different carotenoid precursors and intermediates [59]. The conclusions of this study were: (1) a high linear correlation between β-carotene, β-cryptoxanthin and cryptocapsin was observed, suggesting that these carotenoids were involved in the same metabolic route and that they might be intermediaries in the same pathway; and (2) a high negative correlation between the levels of these three carotenoids and xanthophylls (capsanthin, capsorubin, neoxanthin, mutatoxanthin, luteoxanthin and cryptoflavin) was observed, indicating that the synthesis of these compounds during maturation was paralleled by a decrease in β-carotene, β-cryptoxanthin and cryptocapsin, which are potential precursors in *Capsicum* carotenoid metabolism. Likewise, it was suggested that β-carotene, β-cryptoxanthin and zeaxanthin were violaxanthin and antheraxanthin precursors, which in turn were the precursors for capsorubin and capsanthin, respectively. β-cryptoxanthin is the β-cryptoxanthin epoxide precursor (Figure 2). Based on these results, a carotenoid pathway in *Capsicum* was proposed (Figure 2) [101,102].

A series of articles were published describing the isolation and characterization of enzymes that are involved in the carotenoid biosynthetic pathway in chili peppers as well as genetic aspects of this pathway. The isopentenyl pyrophosphate isomerase (IPI) and GGPS enzymes were first isolated and purified to homogeneity from chili pepper chromoplast stroma using aminophenethyl pyrophosphate-affinity chromatography [67]. IPI was revealed to be a monomeric enzyme with a molecular weight of 33,500 ± 500 Da and a *K*_m_ of 6 μM for isopentenyl pyrophosphate. GGPS was found to be dimeric with a native molecular weight of 74,000 ± 2000 Da resulting from the association of two identical subunits with molecular weights of 37,000 + 1000. GGPS catalyzes the prenyl transfer reaction using isopentenyl pyrophosphate (*K*_m_ = 3 μM) and either dimethylallyl pyrophosphate (*K*_m_ = 0.95 μM), geranyl pyrophosphate (*K*_m_ = 1 μM) or farnesyl pyrophosphate (*K*_m_ = 1.2 μM). It appears that GGPS is non-covalently bound but is always associated with IPI or the PSY enzyme complex in the chromoplast stroma of *Capsicum* [67]. A corresponding cDNA was further identified, isolated using specific antibodies, and sequenced [68]. The cloned cDNA encoded a precursor of 369 amino acids with a transit peptide of approximately 60 amino acids. GGPS was exclusively immunolocalized in plastids. Expression analysis demonstrated that this gene was strongly induced during the chloroplast to chromoplast transition in ripening chili pepper fruits and correlated with an increase in the enzyme activity. The *GGPS* gene was cloned, and the nucleotide sequence indicated an open reading frame of 369 codons that was identical to the cDNA without any interruption by introns, and Southern analysis of *C. annuum* genomic DNA suggested the presence of a single copy [103]. *GGPS* gene expression was induced in chili pepper fruit tissues by wounding [104].

The formation of phytoene from the condensation of two GGPP molecules, a two-step reaction catalyzed by PSY, is the first step in plant carotenoid biosynthesis (Figure 2) [79]. The site of phytoene biosynthesis was localized exclusively to the plastid compartment [105]. This enzyme was isolated and purified to homogeneity by affinity chromatography from the chromoplast stroma of chili pepper fruits and was further characterized, exhibiting a monomeric structure with a molecular size (*M*_r_) of 47,500 Da and a Michaelis-Menten kinetics value of 0.30 μM for geranylgeranyl pyrophosphate. The enzymatic activity required Mn^2+^ and was inhibited by inorganic pyrophosphate and hydroxyphenylglyoxal (an arginine-specific reagent) [79]. DNA sequence analysis of the *PSY* genes from seven orange fruit chili pepper varieties revealed the length of the gene to be 2849 bp with six exons, five introns, and a predicted protein consisting of 420 amino acids [61].

PDS in chili peppers carries out a two-step desaturation reaction of phytoene, which is subsequently converted into phytofluene and ζ-carotene, the first visible carotenoid (Figure 2). PDS was isolated from *Capsicum annuum* chromoplast membranes and exhibited a 56 ± 2 kDa molecular weight, an apparent optimum pH of 7.2–8, and a *K*_m_ of 7 and 4 μM for phytoene and phytofluene, respectively. Additionally, FAD bound to the protein [81]. Antibodies raised against purified PDS were used to isolate a full-length cDNA (2000 bp) that corresponded to an open reading frame of 582 codons and to a 65 kDa protein; the deduced primary structure confirmed a characteristic dinucleotide-binding site and an 81% identity with a PDS from soybean (*Glycine max*). Heterologous expression of a *PDS* cDNA in *E. coli* permitted the production of a recombinant desaturase, which exhibited the same properties as chromoplast PDS. Analysis of *PDS* mRNA levels showed expression in seedlings, young expanding leaves, senescing leaves and fruits. Low expression in green fruits and a slight increase before the detection of carotenoid synthesis was observed, and levels then remained constant through ripening. However, PDS activity and protein levels increased significantly during the transition from chloroplast to chromoplast [81].

The two-step desaturation reaction conversion of ζ-carotene into neurosporene and lycopene is catalyzed by ZDS, a chromoplast membrane-bound enzyme (Figure 2). This enzyme was purified and characterized after heterologous expression of a *ZDS* cDNA from *Capsicum annuum* in *E. coli* [84,106]. The deduced sequence of the *ZDS* cDNA encoded a mature protein of approximately 59 kDa, which is synthesized as a precursor with a transit peptide for plastid localization [84]. ZDS exhibited a monomeric structure with lipophilic quinones as cofactors (decyl-plastoquinone or decyl-ubiquinone increased its activity) [106]. Evidence of dimerization was established, but no differences in activity between the monomeric and dimeric forms were observed. Very similar *K*_m_ values were found for ζ-carotene (8.4 μM) and neurosporene (9.0 μM), indicating that both carotenes are converted at similar rates in the first and second desaturation steps [106].

As it was previously mentioned, the reactions catalyzed by PDS and ZDS have been proposed to be linked or coupled to a plastid terminal oxidase. A *PTOX* gene cDNA (1387 bp) was cloned from chili pepper fruits (*C. annuum*) [107]. Genomic analysis suggested the presence of a single gene copy, and the expression correlated with that of *PDS* and *ZDS* genes during fruit ripening.

Lycopene is the precursor of cyclic carotenoids such as β-carotene, an important component of the reaction centers and antenna of the photosynthetic apparatus. Lycopene is also a substrate for the biosynthesis of various other carotenoids (xanthophylls, zeaxanthin, antheraxanthin, violaxanthin and neoxanthin). Abscisic acid synthesis occurs via β-carotene and the derived carotenoids (Figure 2). β-carotene is the most important precursor of vitamin A for human and animal food. Carotenoid cyclization reactions are associated with chromoplast membranes [96], and LCY-B was isolated from *C. annuum* fruit chromoplast membranes [108]. The membrane-solubilized enzyme was capable of incorporating labeled lycopene into β-carotene but not into α-carotene. No NADP or FAD was required as a cofactor, and sulfhydryl reagents severely inhibited its activity, indicating that SHgroups were involved in the cyclization process. A cDNA encoding the enzyme catalyzing the cyclization of lycopene to β-carotene (LCY-B) was also isolated from *Capsicum annuum* fruits and expressed in *E. coli* [109]. The deduced amino acid sequence of the cloned *LCY-B* cDNA was 498 residues long with a calculated molecular weight of 55.6 kDa for the precursor polypeptide, including the transit peptide for chloroplast/chromoplast targeting, whereas the molecular weight of the mature protein was approximately 50 kDa. A potential dinucleotide-binding site was also observed at the NH_2_ terminus. Alignment with the CCS sequence revealed an identity of 55% and a similarity of 72%. *LCY-B* was expressed in young and senescent leaves and showed constitutive expression during chili pepper fruit development. Recently, sequence analysis of *LCY-B* genes from seven orange fruit chili pepper varieties indicated a size of 1495 bp and the absence of introns in the genomic DNA for all seven varieties [61]. The predicted protein was 499 amino acids long.

With regard to the synthesis of zeaxanthin from β-carotene, BCH is the enzyme that together with a CYP97A enzyme catalyzes this two-step reaction (Figure 2). A *BCH* type gene sequence analysis (*CRTZ-2*) from the genomic DNA of seven orange fruit varieties indicated the gene to be 2150 bp in size; the coding region was 2026 bp (316 amino acids) and contained six introns and seven exons [61].

ZEP is the enzyme that catalyzes the transformation reaction of zeaxanthin into antheraxanthin and later into violaxanthin (Figure 2). ZEP also catalyzes the conversion reaction of β-cryptoxanthin to β-cryptoxanthin epoxide. A *ZEP* cDNA from chili pepper (*C. annuum*) was cloned using a *Nicotiana plumbaginifolia* cDNA probe, and the corresponding enzyme was over-expressed in *E. coli* and purified [102]. This enzyme specifically acted on the β-ring of xanthophylls such as β-cryptoxanthin, zeaxanthin and antheraxanthin in the presence of numerous carotenoid substrates possessing є- (α-carotene and lutein) and β-rings (β-carotene, β-cryptoxanthin and antheraxanthin). Purified ZEP incubated with zeaxanthin in the presence of the stromal proteins ferredoxin or rubredoxin produced violaxanthin and antheraxanthin, as determined by HPLC [102]. Similarly, when ZEP was incubated with β-cryptoxanthin, the reaction product was β-cryptoxanthin-5,6-epoxide. These results indicate that ZEP catalyzes the following reactions: (1) β-cryptoxanthin to β-cryptoxanthin epoxide; (2) zeaxanthin to antheraxanthin; and (3) antheraxanthin to violaxanthin (Figure 2). The deduced peptide sequence showed a characteristic FAD binding domain, and the recombinant enzyme (65 kDa) required NADPH-reduction power to be transferred to the epoxidase via ferredoxin for the cyclohexenyl-carotenoid epoxidation. This gene was found to be upregulated in oxidatively stressed seedlings and during the chloroplast-chromoplast differentiation process in chili pepper fruits.

CCS is the enzyme that synthesizes the final carotenoid products (capsanthin and capsorubin) in red chili pepper fruits (Figure 2). This enzyme catalyzes the transformation of the 5,6-epoxycarotenoids antheraxanthin and violaxanthin into capsanthin and capsorubin, respectively. The *in vitro* conversion of labeled violaxanthin and antheraxanthin into capsorubin and capsanthin by a chromoplast-enriched fraction from chili pepper fruits was demonstrated early on [98,99]. The capsanthin-capsorubin synthase name was proposed due to its bi-functionality. CCS was isolated and purified from *Capsicum annuum* chromoplasts [101]. The purified enzyme was found to be a 50 kDa monomer. Cloning of a *CCS* cDNA and the expression pattern analysis in leaves and fruits during several ripening stages and in different tissues demonstrated that the *CCS* gene was specifically expressed during chromoplast development in mature chili pepper fruits. The *CCS* gene was exclusively expressed in fruits that accumulated keto-carotenoids but not in impaired mutants during this biosynthetic step (yellow-fruited mutants lacking the red pigments capsanthin and capsorubin) [101]. *CCS* gene expression was induced in chili pepper fruits by wounding [104]. Promoter sequence analysis (−588 bp) of the *CCS* gene revealed the presence of *ABA* (abscisic acid-responsive), *ELI* (elicitor-responsive), *HD* (homeodomain) and *WUN* (wound-responsive) *cis*-acting elements [104]. When this promoter was fused to *GUS-*encoding sequences, expression was induced by ROS (reactive oxygen species), indicating that *CCS* gene expression is controlled by ROS. CCS also showed LCY-B activity (catalyzing the conversion of lycopene into β-carotene) when a *CCS* cDNA was expressed in *E. coli* [109]. A proposed molecular model for carotenoid channeling in *C. annuum* fruits suggested that massive and specific channeling of linear carotenoids into the β-carotene pathway in red *C. annuum* fruits is due to the concomitant action of LCY-B and CCS on β-cyclic carotenoids [109]. This model takes into consideration the fact that *LCY-B* gene is not upregulated during fruit ripening and does not require negative regulation of α-carotene biosynthesis. The *LCY-B* gene and *CCS* sequences from *C. annuum* share 55% identity, which suggests that both genes originated from a common ancestral gene [109]. The *CCS* gene was found to be 1494 bp long with no introns and a predicted size of 499 amino acids when genomic DNA from six orange chili pepper fruit varieties was analyzed [61].

As it was previously mentioned, violaxanthin can be converted into capsorubin in chili peppers, but this carotenoid can also be potentially transformed into xanthoxin, a precursor of ABA, by the action of NCED, a carotenoid cleavage dioxygenase (CCD). Until now, no reports on this type of enzymes in chili peppers have been published, but just a 1776 bp sequence (Y14164) has been deposited in the NCBI.

## 6. Regulation of Carotenoid Biosynthesis in *Capsicum*

Plants have evolved complex regulatory mechanisms to control carotenoid biosynthesis and its accumulation. In general, the carotenoid concentration represents a steady state resulting from a combination of biosynthesis and degradation [110]. Carotenoid biosynthesis genes have been cloned from several plants, such as *Arabidopsis*, tomato, chili pepper, daffodil and marigold. These genes are highly regulated and directly connected to plant phenotype color [111]. It has been shown in tomato fruits and marigold petals that carotenoid accumulation is mainly controlled by the transcriptional regulation of carotenoid biosynthetic genes [112–114]. In the case of *Capsicum*, carotenoid-biosynthesis regulation at the gene and enzyme levels is not fully understood. It is well known that the total carotenoid content is quite diverse among *Capsicum* species (*C. baccatum*, *C. chacoense*, *C. chinense*, *C. frutescens* and *C. annuum*) [115]. *Capsicum annuum* lines very often exhibit the highest carotenoid content, ranging between 390 and 16,600 μg/g (d.wt.) [115]. These data suggest that there may be several regulation steps in carotenoid biosynthesis, depending on the genotype.

Several approaches have been used to study carotenoid biosynthesis regulation, such as genetic analysis of the fruit color trait, characterization of mutant plants defective in the production of various carotenoids, expression analysis of structural biosynthetic genes, and over-expression of structural genes.

Fine genetic control of carotenoid production is responsible for the type and quantity of carotenoid accumulation in chili pepper fruits. It has been established that mature chili pepper fruit color is determined by three loci (three independent pairs of genes): *c1*, *c2* and *y* [116]. Furthermore, using RFLP and specific-PCR to analyze DNA sequences and thus determine polymorphisms for the *CCS* gene in F_2_ progeny (this filial was derived from a cross between red and yellow fruit-producing chili pepper plants) showed that *CCS* completely segregated with the red fruit color and that locus *y* (encoding for *CCS*) controlled the red character [117]. These results support the hypothesis that the yellow chili pepper fruit color phenotype might be the result of a *CCS* gene deletion. A co-segregation of the *y* locus and *CCS* in populations generated from crosses between chili pepper plants bearing red × white and red × yellow fruits indicated the correspondence of the two genes [118]. Similarly, the *CCS* gene completely co-segregated with fruit color in the F_2_ population, as assessed by a PCR polymorphism analysis of the *CCS* gene and the generation of a TLC separation carotenoid profile, suggesting that *CCS* determined the chili pepper fruit color by altering the carotenoid pattern.

A genetic map was established using RFLP and AFLP markers and an F_2_ population derived from an interspecific cross between *Capsicum annuum* cv. TF68 and *Capsicum chinense* cv. Habanero [119]. The TF68 ripe fruit was red and its Habanero counterpart was orange. The red color was dominant over the orange in the F_1_ population. To identify the gene(s) or marker(s) tightly linked to the red/orange locus, several candidate genes involved in the carotenoid biosynthetic pathway, including *GGPS*, *PSY*, *PDS*, *LCY-B* and *CCS*, were examined. The results showed that only the *PSY* gene completely co-segregated with the color in the F_2_ population, suggesting that this gene corresponded to the *c2* locus responsible for the accumulation of red color [119]. Similarly, the *PSY* locus was associated with the content of individual pigments such as capsanthin, capsorubin and zeaxanthin. These results strongly suggested that carotenoid levels in fruits were determined by the composition of the *PSY* allele. In the same way, QTL and HPLC analysis of F_2_ lines demonstrated that *PSY* was the locus responsible for fruit color in ripe red peppers (*Capsicum* spp.) [119]. The *PSY* locus was not linked to the other candidate *CCS* gene, which implies that *PSY* and *CCS* segregated independently. Therefore, the orange-color phenotype might be the result of reduced PSY activity rather than a lack of red pigment. Thus, this enzyme was proposed to be the rate-limiting step of carotenoid biosynthesis [119]. More recently, a splicing mutation in the *PSY* gene was demonstrated to cause orange coloration in Habanero (*C. chinense*) chili pepper fruits [120]. This mutant exhibited a recessive *c2* homozygous allele with a point mutation at the splice acceptor site of the fifth intron of the *PSY* gene, which causes both a frame-shift and a premature translational termination and is responsible for the orange fruit color.

Borovsky *et al.* [121] described an EMS-induced chili pepper mutant affected in the *CHY2* gene, which encodes a β-carotene hydroxylase. This mutant produced and accumulated orange carotenoids (β-carotene) in the fruits instead of the red ones of the red-fruited progenitor “Maor”. Mutation was caused by an *A*^709^ to G transition in the *CHY2* gene. These authors hypothesized that *CHY2* controls the orange mutation in chili pepper. *chy2* mutant also exhibited changes in volatile compounds (higher norisoprenoid levels).

When *CCS*-defective mutants were characterized the presence of a 240 bp deletion at the 5′ end in the coding region of the *CCS* gene from yellow-fruited lines of *Capsicum* was demonstrated, indicating that this gene determines the red color in the pericarp of chili pepper fruits [118]. A deletion in the upstream region of the *CCS* gene in orange-fruit pepper plants was also found [120]. Using Southern hybridization and sequencing analysis, a 211 bp sequence of the downstream region of the gene was detected in plants with orange fruits, while no transcripts of the *CCS* gene were demonstrated by RT-PCR in mature orange fruits [122]. Therefore, the orange color of chili pepper fruits was the consequence of a *CCS* gene deletion. *C. chinense* varieties Y2 (PI 800065) and Y3 (PI 164918), which do not accumulate capsanthin in the fruits, possess the encoding and promoter regions of the *CCS* gene in their genomes [111]. Two structural mutations in Y2 and Y3 chili peppers were detected by multiple alignment analysis of the nucleotide sequences from the *CCS* coding regions. One of these mutations resulted in a frame-shift, causing early termination of translation via an 8 bp insertion at position 1431 of the gene in Y2. The *CCS* mutation in Y3 produced a premature stop-codon via a single base change at position 599. The Y2 chili pepper could generate a truncated CCS protein of 495 amino acids, while the Y3 pepper could generate a truncated CCS protein of 119 amino acids. This PTC phenomenon can be explained by a mechanism called “nonsense-mediated mRNA decay” [123], in which mRNAs harboring premature translation-termination codons decay rapidly because the resulting *C*-terminally truncated proteins can function as dominant negative inhibitors of the full-length protein. Likewise, *CCS* mutations are a key example of carotenoid biosynthesis regulation in ripe yellow chili pepper fruits [111]. Similarly, it was observed that in *C. baccatum*, *C. chinense*, *C. annuum* and *C. pubescens* red pepper fruits, the induction of *PSY*, *PDS* and *BCH*, in addition to *LCY-B*, *VDE* and *CCS* genes, during ripening was required to accumulate high total carotenoid levels; therefore, the expression levels of carotenoid biosynthetic genes are the critical for the accumulation of high total carotenoid levels in chili pepper fruits [111]. Recently, a *CCS* variant gene (*ccs-3*) was described in an orange-fruited chili pepper variety (Fogo) that does not accumulate capsanthin and capsorubin [61]. This *ccs-3* variant gene contained an early termination stop codon due to the deletion of a cytosine at nucleotide position 1283, which caused a frame-shift in the coding sequence, thus giving rise to a polypeptide product of 423 amino acids. Li *et al.* [124] described a new *ccs* chili pepper (*C. annuum*) variant in the yellow fruit line CK7 with a premature stop codon derived from a C to G change (1095 bp downstream of the start codon), and also a downstream frame-shift caused by a 1 bp nucleotide deletion (1265 bp downstream of the start codon). This variant exhibited positive expression of the mutant CCS protein.

Carotenoid biosynthesis and accumulation is influenced by different factors. The role of reactive oxygen species (ROS) as regulators of *in vivo* carotenoid biosynthesis was tested in chromoplasts from mature-green stage bell pepper fruits (*C. annuum* cv. Yolo Wonder) [104]. The addition of ROS-inducing compounds such as menadione, tert-butylhydroperoxide or paraquat and pro-oxidants such as diamide or buthionine sulfoximine to green pericarp discs from chili pepper fruits rapidly and dramatically induced the simultaneous expression of multiple carotenogenic gene mRNAs. Menadione induced *GGPS* and *CCS* gene expression, and tert-butylhydroperoxide triggered *GGPS*, *PSY*, *PDS*, *ZEP* and *CCS* gene expression but not *LCY-B*. Pericarp disks treated with paraquat showed a strong induction of *CCS* mRNA accumulation, and those treated with diamide and buthionine sulfoximine exhibited over-expression of *GGPS* and *CCS*. Similarly, down-regulation of catalase by amitrole (which inhibits catalase activity) induced the expression of carotenogenic gene mRNAs (*GGPS*, *PSY*, *PDS*, *LCY-B*, *ZEP* and *CCS*), leading to the synthesis of capsanthin in excised green pericarp discs. These results suggested that ROS acted as a novel class of second messengers that mediated intense carotenoid synthesis during chromoplast differentiation. As previously mentioned [104], characterization of the *CCS* promoter revealed the presence of *ABA* (abscisic acid-responsive), *ELI* (elicitor-responsive), *HD* (homeodomain) and *WUN* (wound-responsive) *cis*-acting elements, suggesting the possibility of *CCS* expression regulation by all of these factors.

The light-dark regulation of carotenoid biosynthesis in chili pepper (*Capsicum annuum*) leaves has been investigated [110]. The contribution of carotenoid biosynthesis to total carotenoid leveles was studied by treating chili pepper plants with 0.1 mmol/l norflurazon (NFZ; an herbicide that blocks carotenoid biosynthesis by inhibiting PDS leading to an accumulation of phytoene) under light and dark conditions. Herbicide-treated leaves from green chili pepper plants exhibited a phytoene concentration of 1.53 mg/g d.wt. under a light period of 280 μmol m^−2^s^−1^ for 48 h. However, when NFZ was applied to plants grown in dark, no accumulation of phytoene was observed. The amount of cyclic carotenoids was approximately the same with and without NFZ. These results indicated that carotenoid biosynthesis was completely blocked when herbicide-treated plants were transferred to dark conditions. Additionally, it was observed that transcripts from all analyzed genes decreased under dark conditions. For instance, *ZDS*, *PTOX* (plastid terminal oxidase) and *LCY-B* transcripts in dark-grown plants were 19%, 24% and 44%, respectively, of their particular levels observed in light-grown plants. With regard to *PSY* and *PDS* transcript levels, dark-treated plants exhibited less than 10% of the levels in their light-grown counterparts. The very low *PSY* transcript levels in non-illuminated plants coincided quite well with the absence of phytoene accumulation in dark NFZ-treated plants. Consequently, the down-regulation of *PSY* appeared to be the central switch in shutting down carotenoid biosynthesis in the dark.

In some cases, the function of a specific gene can be demonstrated by heterologous over-expression. When the *CCS* gene was over-expressed in *Nicotiana benthamiana* plants by means of a viral RNA vector (*Tobacco mosaic virus*) bearing the *CCS* cDNA under the Tobamovirus subgenomic promoter, *CCS*-infected plants developed an orange color phenotype in their leaves as a consequence of the accumulation of high capsanthin levels (up to 36% of total carotenoids) [125]. Interestingly, the capsanthin was not esterified. This carotenoid accumulation was associated with thylakoid membrane distortion and reduction of grana stacking. The high capsanthin accumulation was balanced by a decrease in xanthophyll content, suggesting that a subtle auto-regulatory system acts to control carotenoid biosynthesis and accumulation in the chloroplast.

Carotenoid biosynthesis is a multi-step (multi-gene) pathway that theoretically must be regulated in a very fine and coordinate way. In general terms, biosynthetic complex pathways are regulated by transcription factors, which coordinate the expression of the participating genes. In the case of regulation of the carotenoid biosynthetic pathway, current understanding of regulation at the transcription level is very limited. Recently, a quantitative comparative expression analysis of the *PYS*, *LCY-B*, *BCH* (*CRTZ-2*) and *CCS* genes in orange-fruited chili pepper cultivars (*C. annuum*) showed a particular carotenoid accumulation profile and also a characteristic expression pattern for each chili pepper type [126]. In one cultivar with the *ccs-3* mutant allele (Fogo), *CCS* gene transcripts were detected, but no CCS enzyme was produced, whereas in two other cultivars (Orange Grande and Oriole) with four wild-type genes, no *CCS* transcripts were recorded, and no capsanthin or capsorubin was produced. In the case of “Canary”, this cultivar expressed the four wild-type genes, but no CCS enzyme was produced, and consequently no red carotenoids accumulated. These results suggest that non-structural genes (potentially transcription factors) might regulate carotenoid biosynthesis.

## 7. Future Research on Chili Pepper Carotenoid Biosynthesis

Carotenoid biosynthesis and their accumulation in *Capsicum* fruits is a broad field of study. Chili pepper fruits contain a wide variety of carotenoid pigments, which comprise a large amount of structural diversity, that are almost exclusively found in *Capsicum* members. For instance, two new carotenoid pigments in *C. annuum* fruits were reported [64]. Moreover, not all carotenoid biosynthesis pathways in chili pepper fruits are completely known and understood, and more research is thus needed to elucidate these carotenoid pathways, including those that produce minor carotenoids, at both the enzymatic and genetic levels. The central role of carotenoids in plant development and adaptation is of paramount importance, as their biosynthesis is coordinated with other developmental processes such as plastid formation and flower and fruit development [3]. Unfortunately, the regulation of carotenoid biosynthesis in *Capsicum* at the gene and enzyme level is currently poorly understood. As of now, only a few studies of transcription factors or other genes that impose global regulatory functions on carotenoid metabolism in plants have been described [74]. Only two types of transcription factors (RAP2.2 and PIFs) have been identified that directly interact with the *Arabidospsis* PSY promoter [127,128]. To our knowledge, no reports of transcription factors that regulate carotenoid biosynthesis in *Capsicum* have been revealed so far. As a result, it is imperative to focus more research on this specific genetic issue to unravel carotenoid production in chili pepper fruits.

Another approach to better understand carotenoid biosynthesis regulation in chili pepper fruits is the study and characterization of promoters from structural biosynthetic genes. Until now, only the *CCS* gene promoter has been partially studied, and some *cis*-element sequences have been reported [81,124]. Characterization of promoter sequences for all of the structural biosynthetic carotenogenic genes should surely reveal possible interactions with transcription factors and also developmental or environmental regulatory elements.

## 8. Plausible Applications

*Capsicum* fruits possess great diversity in terms of color, flavor, pungency and nutritional value. These fruits contain high pigment content including carotenoids and anthocyanins, and vitamins such as C, A and E [32]. It was demonstrated that chili pepper pigments, including carotenoids, possess high antioxidant and anticarcinogenic activities [3,33,129–131]. Due to their high carotenoid pigment content, ripe chili pepper fruits are in high demand in industry, as they are used as colorants for cosmetics and foods [4]. Additionally, some chili pepper fruit carotenoids have provitamin A activity, and so their consumption is beneficial for human and animal health, as both humans and animals do not synthesize carotenoids *de novo* and rely upon the diet as the source of these essential products [3]. Therefore, it is essential to have knowledge about the carotenoid biosynthesis steps and their regulation at the genetic, molecular and enzymatic levels to understand the accumulation process in chili pepper fruits. If those chili pepper carotenoids that show maximal health benefits can be increased, doing so may be beneficial in reducing the incidence of human diseases, including age-related macular degeneration and cancer. Furthermore, carotenogenic genes from *Capsicum* species could be useful to increase the beneficial value of other important crops through genetic engineering approaches [132]. For example, a construct bearing a recombinant *PSY* gene from *Capsicum* and a carotene desaturase (*CrtI*) gene from *Pantoea* under the control of either the β*-conglycinin* gene or *CaMV-35S* promoter was used to transform *Glycine max* via *A. tumefaciens* infection, and the resulting transgenic plants showed a 62-fold increase in total carotenoids in the seeds, of which 77% corresponded to β-carotene [133].

## 9. Conclusions

Carotenoids are responsible for the yellow, orange and red colors of chili pepper fruits. The carotenoid biosynthetic pathway in *Capsicum* chili pepper fruits has been fully established. Mutants that are impaired in some carotenoid structural genes, thus resulting in a great diversity and variety of chili pepper fruit colors, have been characterized to understand carotenoid biosynthesis and regulation. Some carotenoid biosynthetic enzymes have been isolated and characterized, and the genes encoding them have been sequenced, but only in a few cases their promoters have been characterized. No regulatory proteins or their encoding genes that control carotenoid biosynthesis in chili pepper fruits have been described. Information is currently being used to select chili pepper materials with high carotenoid contents. Genetic engineering to improve or to modify the carotenoid content of chili pepper fruits is pending.

## Figures and Tables

**Table 1:** Carotenoid contents in fruits from several *Capsicum annuum* varieties.

Chili pepper type	Ripening stage	Main carotenoids	Total carotenoids	Reference
Red bell pepper	Mature (Red)	Capsanthin (34.7%), β-carotene (11.6%) and violaxanthin (9.9%)	284 and 127 mg per kg f.wt. (as β-carotene) (two lots of peppers)	[27]
Green bell pepper	Immature (Green)	Lutein (40.8%), neoxanthin (15.1%), violaxanthin (13.8%) and β-carotene (13.4%)	10.6, 11.2 and 9.0 mg per kg f.wt. (as β-carotene) (three lots of green peppers)	[52]
Yellow pepper	Immature (Green)	Violaxanthin (34%), antheraxanthin (10.5%), lutein (9.2%) and zeaxanthin (8.5%)	13.2 mg/100 g d.wt.	[53]
	Mature (Orange)	Lutein (37.8%), β-carotene (19.8%) and neoxanthin (5.5%)	488.6 mg/100 g d.wt.	
Black paprika	Immature (Black)	Lutein (28.5%) and zeaxanthin (11.96%)	48.5 mg/100 g of d.wt.	[54]
	Mature (Red)	Capsanthin (42%), zeaxanthin (8%), capsorubin (3.2%) and β-carotene (7%)	3211 mg/100 g d.wt.	
Szentesi Kosszarvú	Immature (Green)	Lutein (31.9%) and β-carotene (11.3%)	11.5 mg/100 g d.wt.	[48]
	Mature (Red)	Capsanthin (29%) and zeaxanthin (15%)	994.7 mg/100 g d.wt.	
*Capsicum annuum* var. *lycopersiciforme rubrum*	Immature (Green)	Lutein (31.6%) and β-carotene (13.7%)	19.6 mg/100 g d.wt.	[55]
	Mature (Red)	Capsanthin (37%), zeaxanthin (8%), and β-carotene (9%)	1297.1 mg/100 g d.wt.	
Sweet peppers	Immature (Green)	Lutein (2.3 mg/100 g f.wt.) and β-carotene (1.7 mg/100 g f.wt.)	5.1 mg/100 g f.wt.	[32]
	Green	Lutein (1.4 mg/100 g f.wt.) and β-carotene (2.1 mg/100 g f.wt.)	4.9 mg/100 g f.wt.	
	Immature (Red)	β-carotene (1.9 mg/100 g f.wt.) and zeaxanthin (2.9 mg/100 g f.wt.)	9.5 mg/100 g f.wt.	
	Red	β-carotene (4.3 mg/100 g f.wt.) and capsanthin (19.9 mg/100 g f.wt.)	45.6 mg/100 g f.wt.	
Ancho, guajillo and mulato	Ancho (Mature)	β-carotene (20.9%), and violaxanthin (14.5%)	7.5 mg/100 g d.wt.	[56]
	Guajillo (Mature)	β-carotene (17.9%), violaxanthin (13.2%)	6.8 mg/100 g d.wt.	
	Mulato (Mature)	Violaxanthin (22%) β-carotene (14.9%)	7.2 mg/100 g d.wt.	
